# Breakthrough SARS-CoV-2 Omicron Variant in Individuals Primed with Heterologous Vaccines Enhances Inhibition Performance of Neutralizing Antibody to BA.2 Parental Lineage

**DOI:** 10.3390/vaccines11071230

**Published:** 2023-07-11

**Authors:** Jidapa Szekely, Piyawut Swangphon, Natthaphon Nanakorn, Panuttha Chaimuti, Teerapat Nualnoi, Paweena Wongwitwichot, Namchoke Somapa, Denpong Somapa, Theerakamol Pengsakul

**Affiliations:** 1 Faculty of Medical Technology, Prince of Songkla University, Hat Yai 90110, Thailand; piyawut.s@psu.ac.th (P.S.); natthaphon.s@psu.ac.th (N.N.); 2 Immunology and Virology Unit, Department of Medical Technology and Clinical Pathology, Hat Yai Hospital, Hat Yai 90110, Thailand; 3 Department of Pharmaceutical Technology, Faculty of Pharmaceutical Sciences, Prince of Songkla University, Hat Yai 90110, Thailand; teerapat.n@psu.ac.th; 4 Department of Pharmaceutical Chemistry, Faculty of Pharmaceutical Sciences, Prince of Songkla University, Hat Yai 90110, Thailand; paweena.w@psu.ac.th; 5 Master Labs Incorporation Co., Ltd., Bangkok 10510, Thailand; 6 Faculty of Environmental Management, Prince of Songkla University, Hat Yai 90110, Thailand; 7 Health and Environmental Research Center, Faculty of Environmental Management, Prince of Songkla University, Hat Yai 90110, Thailand

**Keywords:** COVID-19, neutralizing antibodies, homologous vaccine, heterologous vaccine, Omicron variants

## Abstract

This study aims to analyze the neutralization ability against Omicron parental variants in five clusters of individuals with different Coronavirus disease (COVID-19) immunity backgrounds, including individuals receiving a homologous or heterologous vaccine without prior infection, recovered patients with homologous or heterologous vaccination, and recovery patients without vaccination. Severe acute respiratory syndrome coronavirus 2 (SARS-CoV-2) surrogate virus neutralization assay was performed on serum samples. Spearman correlation analysis showed that the percent inhibition against Omicron B.1.1.529 and BA.2 was significantly related to the period of serum collection (*r* = 0.730 and 0.787, *p* < 0.001, respectively). Very strong correlation between percent inhibition of neutralizing antibody against Omicron B.1.1.529 and BA.2 variants (*r_s_* = 0.973, *p* < 0.001) was also observed. The neutralizing activity of the sera from recovery patients receiving homologous and heterologous vaccine against the wild-type, B.1.1.529, and BA.2 Omicron variants was significantly higher (*p* < 0.001) than that of recovery patients without vaccination. This study robustly showed that the breakthrough SARS-CoV-2 Omicron variant in individuals who received homologous and heterologous vaccines had a high level of neutralizing activity against B.1.1.529 and BA.2 parental lineage of XBB subvariants. Therefore, the next-generation COVID-19 vaccine against emerging variants is needed to improve resilience against ongoing variants, particularly for persons who have never been infected.

## 1. Introduction

Although Coronavirus disease (COVID-19) is no longer considered a global health emergency, the sublineages of parental BA.2 Omicron variant still circulate globally, and COVID-19 can become more dangerous again through mutations. The Omicron variant, which was first detected in November 2021 in South Africa, contains 37 spike protein mutations, which has significantly increased its transmissibility and immune evasion ability, leading to a global rise in breakthrough infections even in countries with high vaccine coverage [1,2,3]. However, a vaccine adapted to the sequence of Omicron variant that contains amino acid sequence similarity in the spike glycoprotein of the receptor-binding domain (RBD), could have broader neutralization potential against all emerging sublineages of the variant [4,5].

In Thailand, COVID-19 has re-emerged since mid-April 2023 because of the country’s liberalization of tourism and the reduction of protection from infection. Once COVID-19 was officially declared endemic in Thailand, the country lifted quarantine requirements for inbound travelers, halted the requirement for regular COVID-19 screening, and the compulsory wearing of face masks applied only to poorly ventilated places and crowded areas. All of these measures cumulatively resulted in a general reduction of protection against COVID-19 infection. The Ministry of Public Health has been instructed to monitor the spread of the XBB.1.16 sub-strain of the Omicron variant. XBB.1.16 is a recombinant or hybrid strain of BA.2.10.1 and BA.2.75 sublineages of the Omicron variant. To enhance immunity in the population, seven COVID-19 vaccines have been approved for prime and boost injections in Thailand, including CoronaVac (Sinovac Biotech Ltd., Beijing, China) inactivated vaccine, Covilo (Sinopharm, Beijing, China) inactivated vaccine, Vaxzevria (AstraZeneca-University of Oxford, Oxford, UK) adenovirus-vector vaccine, Jcovden (Janssen Sciences, Ireland) adenovirus-vector vaccine, Comirnaty (Pfizer-BioNTech Inc., New York, NY, USA) mRNA vaccine, Spikevax (Moderna-NIAID, Cambridge, MA, USA) mRNA vaccine, and Covovax (Serum Institute of India, Pune, India) protein subunit vaccine. Vaccine regimens have no fixed schemes, mostly depending upon availability and preference. Since November 2020, 74.6% of the total population was said to be fully vaccinated using at least two doses (in any combination) of the vaccines listed above by 2 December 2022, while 79.5% were reported to receive at least one dose of the above-listed vaccines [6]. Due to the adverse side-effects of COVID-19 vaccines, including local and systemic side effects [7], the demand for booster doses has been decreasing. This could be of concern regarding the population’s ability to protect themselves from upcoming SARS-CoV-2 variant infection. It is thus of interest to determine whether the population is likely to maintain strong protection against a sub-lineage of BA.2 Omicron variant. Being one of the few countries with various types of vaccines available, Thailand is uniquely positioned to study the effectiveness of homologous versus heterologous vaccine booster regimens against SARS-CoV-2 variants of concern (VOCs) within a single population.

Here, we examined five different groups of individuals with different COVID-19 immunity backgrounds: (1) individuals receiving at least two doses of homologous vaccine without prior infection, (2) individuals receiving at least two doses of heterologous vaccine without prior infection, (3) recovery patients with at least two doses of homologous vaccine, (4) recovery patients with at least two doses of heterologous vaccine, and (5) recovery patients without vaccination. The study was carried out during the Delta wave and the Omicron wave. We evaluated the levels of antibodies against the RBD of the wildtype strain and the neutralizing capability against the Omicron variants, B.1.1.529 and BA.2, compared to the ancestral strain (Wuhan-Hu-1). These results are important for evaluating the relative effectiveness of different COVID-19 immunity backgrounds against Omicron, thereby providing information to enact better public health policies nationwide and globally.

## 2. Materials and Methods

### 2.1. Sample Collection and Study Design

This is a descriptive study, and simple random sampling was used as the sampling method. Two cities with governmental hospital service for COVID-19 vaccination and COVID-19 patient treatment were chosen: Hat Yai (310,460 inhabitants) and Phatthalung (97,489 inhabitants) based on officially available data from the Ministry of Public Health, Thailand [8]. The sample size was estimated assuming the expected 70% frequency of vaccinated Thai population, a 10% margin of error, and a 95% confidence level. With these assumptions, the minimum sample size for each city was 81 subjects, resulting in a total minimum required sample size of 162 subjects. Volunteer recruitment was announced publicly in the study hospitals. The inclusion and exclusion criteria of each study group are described below. Blood sample collection and COVID-19 infection determination of the participants were performed by healthcare professionals in Hat Yai and Phatthalung hospitals. The study protocol was conducted in accordance with the Declaration of Helsinki and Good Clinical Practice principles, and it was approved by the Research Ethics Committee of Hat Yai Hospital (protocol code HYH EC 079-64-02). All participants provided written informed consent.

**Figure 1:**
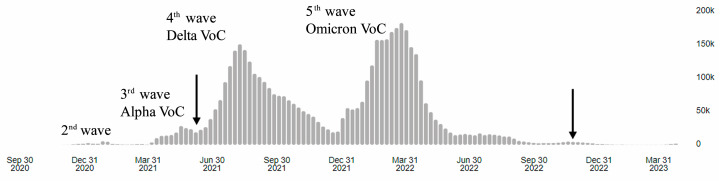
Temporal distribution of confirmed COVID-19 cases in Thailand (official records of the Ministry of Health, Thailand). Arrows indicate the beginning and the end of sample collection in this study [9].

The percentage inhibitions of a neutralizing antibody were assessed using an in vitro surrogate virus neutralization assay. The study was conducted from June 2021 to December 2022 in Songkhla and Phatthalung provinces, Thailand. Serum samples were collected throughout that time period from individuals who received at least two doses of homologous or heterologous COVID-19 vaccinations, called VHo and VHe, respectively. The exclusion criterion of these groups was a history of previous SARS-CoV-2 infection. In addition, two groups of serum samples were obtained from patients with hybrid immunity, including patients infected with SARS-CoV-2, who received homologous vaccination (ReHo), or heterologous vaccination (ReHe). The reference group was serum samples from recovered patients who were infected with SARS-CoV-2 at the beginning of the third wave in early 2021 and never received COVID-19 vaccination (Re). The homologous vaccine schemes in this study include [Astra Zeneca (AZ) × 2,], [BioNTech (PZ) × 2 and × 3], [Sinopharm (SP) × 2], [Sinovac (SV) × 2]. Regardless of dose number, the heterologous vaccine schemes include [AZ + PZ], [SP + AZ], [SP + PZ], [SP + PZ + Moderna (MD)], [SV + AZ], [SV + PZ], [SV + AZ + MD], [SV + AZ + PZ]. The participants’ sera were collected within three months after the full-dose vaccination or infection. The level of anti-SARS-CoV-2 IgG antibodies and % inhibition of neutralizing antibodies from recovery patients were used as reference levels. The status of infection and recovery was confirmed via standard RT-PCR and a professional antigen test for SARS-CoV-2 infection. Asymptomatic and symptomatic individuals with existing medical conditions were required to undergo confirmatory tests for SARS-CoV-2 infection at quick test centers and clinics. Confirmed infections from June 2021 to December 2022 were included in the study, since this was the period of Delta and Omicron dominance in Thailand (Figure 1). The COVID-19 vaccination data recorded on the international COVID-19 vaccination certificate issued by the Thai Ministry of Public Health were provided and written in case record forms by participants.

### 2.2. Quantification of IgG Level

Levels of anti-SARS-CoV-2 IgG antibodies, including neutralizing antibodies which bind to RBD of SARS-CoV-2 spike protein, were quantified by SARS-CoV-2-IgG II Quant assay using the Architect i2000SR instrument (Abbott, Ireland), following the manufacturer’s instructions. This assay is a two-step immunoassay for the qualitative and quantitative determination of IgG antibodies to SARS-CoV-2 in plasma using chemiluminescent microparticle immunoassay (CMIA) technology. The sample, SARS-CoV-2 antigen-coated paramagnetic microparticles, and assay diluent are combined and incubated. The IgG antibodies to SARS-CoV-2 present in the sample bind to the SARS-CoV-2 antigen-coated microparticles. Anti-human IgG acridinium-labeled conjugate is added to create a reaction mixture and incubated. The resulting chemiluminescent reaction is measured as a relative light unit (RLU). There is a direct relationship between the amount of IgG antibodies to SARS-CoV-2 in the sample and the RLU detected by the system optics. The cutoff of the test is 50 arbitrary units per milliliter (AU/mL). Based on the results described in this WHO International Standard study, the mathematical relationship of the Abbott AU/mL unit to the WHO binding antibody units per milliliter (BAU/mL) unit follows the equation: BAU/mL = 0.142 × AU/mL. The results were determined using WHO BAU/mL [10].

### 2.3. SARS-CoV-2 Surrogate Virus Neutralization (sVNT) Assay

The total immunodominant-neutralizing antibodies targeting the viral S protein receptor-binding domain were detected using a SARS-CoV-2 surrogate virus neutralization (sVNT) assay (GenScript, Nanjing, Jiangsu, China), following the manufacturer’s instructions. The sVNT assay detects total immunodominant-neutralizing antibodies targeting the viral spike (S) protein receptor-binding domain of wild type strain Wuhan-Hu-1, Omicron B.1.1.529, or Omicron BA.2 variants of SARS-CoV-2. To mimic the virus-host interaction, the test uses purified RBD from the viral S protein and the host cell receptor ACE2. This RBD-ACE2 interaction can be neutralized by specific neutralizing antibodies in human sera, in the same manner as in conventional virus neutralization test (cVNT) or pseudovirus neutralization test (pVNT). This assay is an alternative to a live virus assay for detecting neutralizing antibodies. For the sVNT assay, Horseradish peroxidase (HRP)-conjugated RBD (wild type strain Wuhan-Hu-1, Omicron B.1.1.529 or Omicron BA.2 variants of SARS-CoV-2) was pre-incubated with test serum at a volume ratio of 1:1 for 1 h at 37 °C (final volume of 50 μL), followed by addition into an enzyme-linked immunosorbent assay (ELISA) plate coated with hACE2 for 1 h at room temperature. Unbound HRP-conjugated antigens were removed by five phosphate-buffered saline, 0.05% Tween-20 washes. A colorimetric signal was developed on the enzymatic reaction of HRP with a chromogenic substrate, 3,3′,5,5′-tetramethylbenzidine. An equal volume of TMB stop solution was added to stop the reaction, and the absorbance readings at 450 nm and 570 nm were acquired using a Cytation 5 microplate reader (BioTek, Agilent Technologies, Inc., Santa Clara, CA, USA). Inhibition (%)  =  (1 − sample optical density value/negative control optical density value) × 100. The cutoff of % inhibition > 30% was positive [11].

### 2.4. Statistical Analysis

The sample size for this study was based on practical considerations rather than statistical power calculations. Statistical analyses were performed using SPSS 22.0 software (SPSS, Inc., Chicago, IL, USA). Correlation between antibody levels and % inhibition against each variant and correlation between % inhibition against different variants were calculated using Spearman’s Rank Correlation. The independent-samples *t*-test was conducted to compare % inhibition against WT and Omicron variants between five different groups. The statistically significant difference of antibody levels and % inhibition against WT and Omicron variants between and within five groups was determined using one-way ANOVA. *Post hoc* multiple comparisons of independent variables were done by the Bonferroni *post hoc* test.

## 3. Results

### 3.1. Sociodemographic Characteristics

A total of 205 participants were recruited in this study, 30.7% males and 69.3% females, with an age range of 18–71 years (Table 1). Of these 205 participants, 25 were recovery patients with no vaccination (Re), 46 and 47 were volunteers with no previous history of SARS-CoV-2 infection but recipients of at least two doses of homologous (VHo) and heterologous (VHe) vaccination, respectively; 16 and 71 participants were recovery patients who had received full doses of homologous (ReHo) and heterologous vaccination (ReHe), respectively. The difference in gender was not statistically significant between the five groups, but the difference in age was statistically significant (*p* = 0.001). In the group recovering from COVID-19, the age range was 18–65 years. The ReHo and ReHe groups age range was 18–68 and 20–56 years, respectively. In addition, the age range of VHo and VHe participant groups was 18–71 and 21–59 years, respectively. Participants with heterologous vaccination were relatively young, while recovery patients were relatively old.

**Figure 2:**
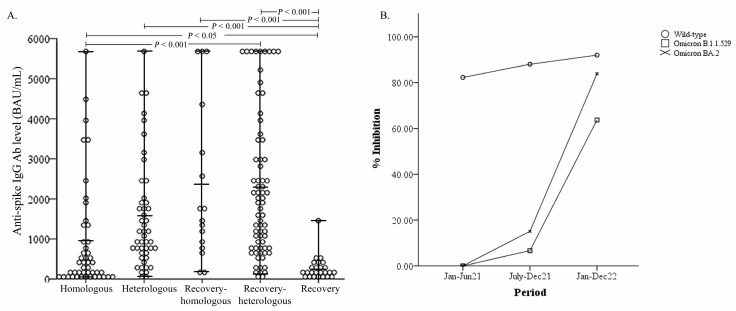
Distribution of the level of anti-spike IgG in five clusters in this study, and distribution of percent inhibition of neutralizing antibody (Ab) against variants in different periods of serum collection. (**A**) Anti-spike IgG level against wild-type SARS-CoV-2 median values with interquartile ranges (IQRs) are shown as horizontal bars. Multiple comparisons of independent variables were done by the Bonferroni *post hoc* test. (**B**) Percent inhibition of neutralizing Ab against wild-type, Omicron B.1.1.529 and BA.2 variants in different periods of serum collection.

In terms of data analysis, the period of serum collection was divided into two distinct periods: the Delta VoC wave and the Omicron VoC wave (Figure 1). The distribution of IgG antibody levels against wild-type SARS-CoV-2 in five clusters of the participants is shown in Figure 2A, while the percent inhibition of neutralizing antibodies against wild-type SARS-CoV-2, Omicron B.1.1.529 (original Omicron variant) and BA.2 Omicron subvariant (a parental lineage of the Omicron XBB variants) between the period of serum collection is shown in Figure 2B.

The results of Spearman correlation analysis showed a strong correlation between % inhibition against Omicron B.1.1.529 and BA.2 and the period of sample collection: the closer the serum collection to the Omicron VoC wave, the higher the neutralizing antibody level against both Omicron variants (*r* = 0.730, *p* < 0.001 and *r* = 0.787, *p* < 0.001, respectively). Furthermore, the distribution of the antibody levels against the wild-type virus between the five study groups is shown in Figure 2A. Meanwhile, % inhibition of antibody against wild-type SARS-CoV-2 remained at high levels (Figure 2B). Multiple comparisons of independent variables by the Bonferroni *post hoc* test showed the antibody IgG levels of VHo, VHe, ReHo and ReHe groups were significantly higher compared to the Re group (Figure 2A). Percent inhibition of neutralizing antibodies against wild-type, Omicron B.1.1.529, and Omicron BA.2 in the vaccine scheme of individuals with prior infection is shown in Supplementary Table S1, and those without evidence of infection are shown in Supplementary Table S2.

### 3.2. Antibody Levels and Neutralizing Ability

**Figure 3:**
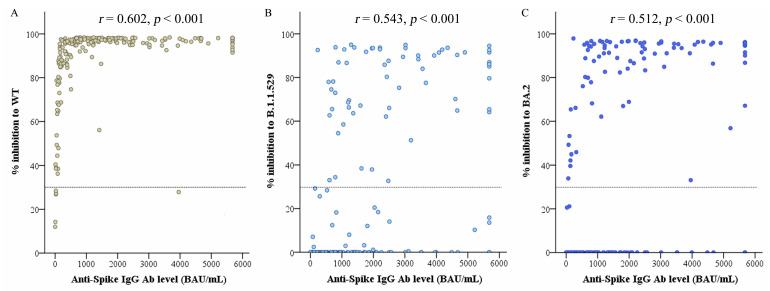
Correlation between anti-spike antibody level against wild-type SARS-CoV-2 and percent inhibition of neutralizing Ab against SARS-CoV-2 variants. Anti-spike IgG level and percent inhibition of neutralizing Ab against (**A**) wild-type, (**B**) Omicron B.1.1.529 and (**C**) Omicron BA.2 variants. Dotted lines indicate cut-off values.

In the present study, anti-spike IgG levels and neutralizing abilities, in terms of % inhibition of neutralizing antibodies, were compared among five different subject groups (Table 2). The Re participants were used as a reference group. These participants had a mean of anti-spike IgG level of 257.342 BAU/mL, while the mean of antibody level was significantly increased by approximately 3.5-, 5.9-, 9.1-, and 8.8-fold (*p* < 0.001) for VHo, VHe, ReHo, and ReHe group, respectively. The correlations between the anti-spike IgG antibody levels against wild-type SARS-CoV-2 and percent inhibition of neutralizing antibody against wild-type, B.1.1.529 and BA.2 Omicron variants are shown in Figure 3A–C. The neutralizing antibody presenting efficacy against wild-type higher than cut-off value at 30% inhibition was observed even at low levels of IgG antibody ranging from 105.05–5680 BAU/mL. When % inhibition of neutralizing antibody against Omicron variants B.1.1.529 and BA.2 were compared to the level of IgG antibody, two distinct groups of results were observed: those with and without neutralizing antibody against the Omicron variants. Neutralizing antibody presenting efficacy against both Omicron variants at a higher than cut-off value was observed in anti-spike IgG antibody levels ranging from 231.09–5680 BAU/mL, and most of these were from the ReHe group. Negative results for neutralizing antibody against both Omicron variants were observed in persons with antibody levels to wild-type ranging from 12.78–4679.94 BAU/mL. This observation indicates that the level of IgG antibody to wild-type does not correlate with the presence of effective neutralizing antibodies to Omicron variants.

**Figure 4:**
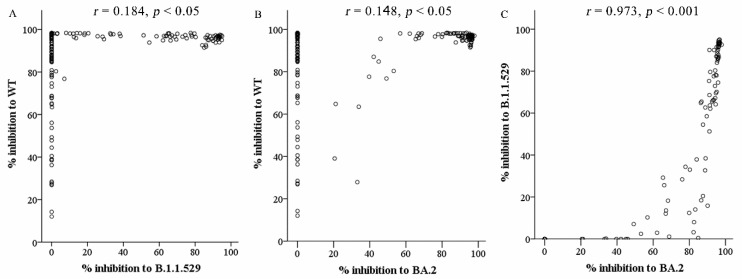
Correlation between the percent inhibition of neutralizing Ab against SARS-CoV-2 variants. (**A**) Percent inhibition of neutralizing Ab between WT and Omicron B.1.1.529, (**B**) between WT and Omicron BA.2 variants, and (**C**) between Omicron B.1.1.529 and BA.2 variants.

The results of Spearman correlation analysis showed a moderate correlation between the antibody levels and the percent inhibition of neutralizing antibody against the wild-type virus (*r_s_* = 0.602, *p* < 0.001), Omicron B.1.1.529 (*r_s_* = 0.543, *p* < 0.001), and Omicron BA.2 variant (*r_s_* = 0.512, *p* < 0.001). Meanwhile, a very weak correlation was discovered between the percent inhibition of neutralizing antibody against wild-type and both B.1.1.529 and BA.2 Omicron variants (*r_s_* = 0.184 and *r_s_* = 0.148, *p* < 0.05, respectively), shown in Figure 4A,B. In contrast, a very strong correlation between percent inhibition of neutralizing antibody against Omicron B.1.1.529 and BA.2 variants (*r_s_* = 0.973, *p* < 0.001) is shown in Figure 4C.

### 3.3. Analysis of Neutralizing Ability

**Figure 5:**
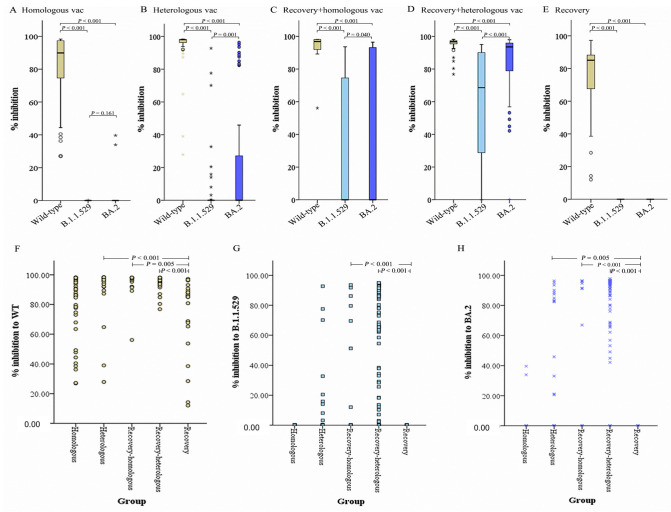
Comparison of percent inhibition of neutralizing Ab against SARS-CoV-2 strains in five different subject groups. (**A**–**E**) Three SARS-CoV-2 strains (wild-type, Omicron B.1.1.529, and Omicron BA.2 variants) were compared in terms of percent inhibition of neutralizing Ab among five different study subjects (homologous vaccinees, heterologous vaccinees, recovering patients with homologous vaccination, recovering patients with heterologous vaccination, and recovering patients, respectively); (**F**–**H**) Five different study subjects were compared in terms of percent inhibition of neutralizing Ab against three viruses (wild-type, Omicron B.1.1.529, and Omicron BA.2 variants), respectively.

Total immunodominant-neutralizing antibodies targeting the viral S protein RBD of wild-type SARS-CoV-2, Omicron B.1.1.529 and BA.2 variants were detected using a SARS-CoV-2 surrogate virus neutralization (sVNT) assay. The neutralizing ability was quantified in terms of the percent inhibition of neutralizing antibody against those three variants. Percent inhibition of the neutralizing antibodies determined in five different study subjects (VHo, VHe, ReHo, ReHe, and Re) was compared. Neutralizing activity of the 46 and 47 sera from homologous and heterologous vaccine recipients (VHo and VHe) against the wild-type, B.1.1.529 and BA.2 Omicron variants were determined. The neutralizing activity of the sera from each group against the B.1.1.529 and BA.2 Omicron variants was significantly reduced as compared to that of the wild-type virus (*p* < 0.001, Figure 5A,B). Of these, 46 and 47 sera from VHo and VHe groups, 95.6% (44/46) and 95.7% (45/47) displayed a neutralizing ability against wild-type virus, respectively. Meanwhile, 100% (46/46) and 91.5% (43/47) of those vaccinees lost the ability to neutralize the B.1.1.529 Omicron variant, and 95.6% (44/46) and 74.5% (35/47) lost the ability to neutralize the BA.2 Omicron variant, respectively. The neutralizing activity of the sera from VHe against the wild-type and BA.2 Omicron variants was significantly higher (*p* < 0.001 and *p* = 0.005, Figure 5F and Figure 5H, respectively) compared to that of the recovery patients. However, sera from VHo group displayed neutralizing activity results not different than that of recovery patients (Table 2).

Similarly, we tested a total of 16 and 71 serum samples obtained from patients who had recovered from COVID-19 and had received full doses of either homologous or heterologous vaccine against SARS-CoV-2 wild-type, called ReHo and ReHe groups, respectively. The results demonstrated that the mean of % inhibition of neutralizing antibodies from ReHo and ReHe groups was 93.25% and 95.60% inhibition against the wild-type virus (Table 2). In contrast, the ability to neutralize the B.1.1.529 and BA.2 Omicron variants of ReHo group was significantly reduced to 30.25% and 39.57%, respectively. However, the ability to neutralize both Omicron variants of sera from the ReHe group was approximately 2.0-fold (*p* = 0.04 and *p* < 0.001) higher than the ReHo group, with 59.59% and 80.9% of inhibition against the B.1.1.529 and BA.2 Omicron variants, respectively (Table 2). Therefore, the neutralizing activity of the sera from ReHo and ReHe groups against the B.1.1.529 and BA.2 Omicron variants was significantly lower than against the wild-type virus (*p* < 0.001, Figure 5C,D). However, the neutralizing activity of the sera from ReHo and ReHe against the wild-type, B.1.1.529, and BA.2 Omicron variants was significantly higher (*p* < 0.001) compared to that of the recovery patients (Figure 5F–H). In addition, the neutralizing activity of sera collected from 25 recovering patients infected with SARS-CoV-2 and who never received any COVID-19 vaccine, was evaluated. The results indicated that the mean of percent inhibition of sera from recovered patients was 73% against wild-type. No neutralizing ability against B.1.1.529, and BA.2 Omicron variants was detected in this group.

### 3.4. Subgroup Analysis

**Figure 6:**
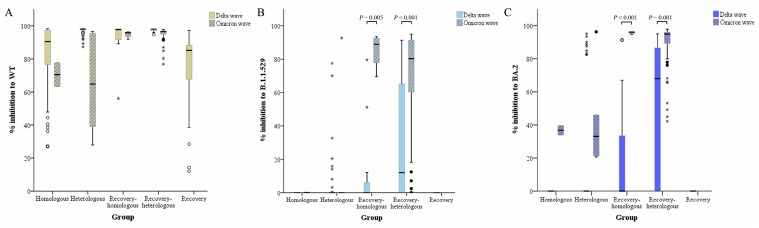
Comparison of percent inhibition of neutralizing Ab against wild-type, Omicron B.1.1.529 and Omicron BA.2 SARS-CoV-2 during Delta wave and Omicron wave in five different subject groups: (**A**) percent inhibition of neutralizing Ab against wild-type, (**B**) Omicron B.1.1.529, and (**C**) Omicron BA.2 variants), respectively.

The sample collection period consisted of two distinct waves, the Delta and Omicron Voc waves, as officially reported in Thailand by the Ministry of Public Health (Figure 1). Neutralizing antibody response to wild-type, B.1.1.529, and BA.2 Omicron strains in five different groups (VHo, VHe, ReHo, ReHe, and Re) was compared during different times of the outbreak wave. Ability level of neutralizing antibody to wild-type strain in these five groups was not statistically significant between different outbreak periods, Delta and Omicron wave, compared within the subject group (Figure 6A). In contrast, the neutralizing activity of the sera from ReHo and ReHe against the B.1.1.529 Omicron variant was statistically significantly higher when collected during the Omicron wave (*p* = 0.005 and *p* < 0.001, Figure 6B). A significant difference was not observed within the VHo and VHe groups between the two waves. Furthermore, the differences in % inhibition of neutralization antibody against the BA.2 Omicron variant were statistically significant between Delta and Omicron waves within the VHo, ReHo, and ReHe groups (*p* < 0.001, Figure 6C), but not within the VHe group. Therefore, the neutralization ability against the wild-type strain was maintained at a high level during the Delta and Omicron waves in all subject groups. The neutralization of Omicron variants was stronger in sera of ReHo and ReHe groups collected during the Omicron wave than during the Delta wave (*p* < 0.001) (Figure 6).

Overall, of 205 participants, 99.02% (203/205) showed anti-spike IgG antibodies against wild-type SARS-CoV-2 above the cut-off value, and the mean of the antibody level increased during the Delta and Omicron waves. The percent inhibition of 93.17% (191/205), 27.80% (57/205), and 39.03% (80/205) of participants showed neutralizing antibodies against the wild-type virus, B.1.1.529 Omicron, and BA.2 Omicron variant above the 50% inhibition, respectively.

## 4. Discussion

The Omicron variant and subvariants have revealed a drastic decrease in antibody reactivity in vaccinated individuals. A COVID-19 booster dose could partially restore the neutralization ability against the Omicron variant, but the extent of neutralization differed drastically among studies. Several booster regimens have been reported to improve the neutralization of emergent SARS-CoV-2 Omicron sub-variants by antibodies elicited in individuals who had previously received two doses of vaccines or had been infected with SARS-CoV-2 [12,13,14,15]. These results suggest the necessity of a booster vaccine for fully vaccinated or convalescent individuals to reduce the risk of symptomatic breakthrough infections by the Omicron variant and ongoing subvariants. It is important to assess the level of protection of vaccinated individuals and of those recovering from SARS-CoV-2 infection with or without vaccination, to provide the information necessary to formulate a travel policy and COVID-19 vaccination schedule to reduce the high risk of SARS-CoV-2 transmission, especially in countries with high levels of tourism. Thailand is one of the countries where a large variety of vaccine regimens have been utilized, which created a unique opportunity to study the effectiveness of homologous versus heterologous vaccine booster regimens against SARS-CoV-2. In this study, we evaluated the levels of antibodies against the RBD of the wild-type strain, the neutralizing capability against the Omicron variants, B.1.1.529 and BA.2, compared to the ancestral strain (Wuhan-Hu-1) in five subject groups.

In agreement with previous studies, we found a near-complete lack of neutralizing activity against Omicron in sera collected during the Delta-dominant period from individuals vaccinated with a homologous vaccine and from convalescent individuals without vaccination. More interestingly, we demonstrated that sera collected from convalescent individuals with heterologous and homologous vaccination during the Omicron-dominant period showed a neutralizing activity significantly higher than those collected during the Delta-dominant period. This finding is similar to that of a study by Gruell et al. (2022), who showed a near-complete lack of neutralizing activity against Omicron in sera from individuals vaccinated with a homologous two-dose mRNA vaccine and from convalescent individuals [16]. In our current study, we found that the homologous vaccine regimen from recovery individuals, which was primed and boosted with mRNA vaccine, showed approximately 80% of inhibition against the BA.2 Omicron variant. Our finding is supported by Yamamoto et al. (2022), who observed a symptomatic Delta breakthrough infection, and three-dose mRNA vaccinees were found to have increased neutralizing antibodies against wild-type, Delta, and Omicron BA.1 [17]. We also found that the heterologous vaccine regimens of the Omicron-breakthrough individuals, which showed approximately 90% of inhibition against the BA.2 Omicron variant, consisted of an inactivated or an adenovirus-vector vaccine as a first or second booster dose, following primary/booster vaccination with mRNA vaccine (Supplementary Table S1). This is corroborated by Zhang et al. (2022), who demonstrated that a significant increase in neutralizing capability (up to 77.85% of neutralization) against Omicron RBD could also be achieved in individuals who received an mRNA booster after two doses of inactivated virus vaccines [15]. This finding is further corroborated by another study by Zuo et al. (2022), who reported that individuals who received an mRNA booster after a two-dose regimen of inactivated virus vaccine showed a 14-fold increase in antibodies that can bind the RBD of Omicron [18]. Regardless of vaccine type, homologous and heterologous booster vaccines will increase protective efficacy against symptomatic SARS-CoV-2 infection [19].

Since the previously dominant Omicron variant and the now-dominant XBB subvariant are the variants that have attracted the most extensive internationally focused attention, developing a vaccine effective against upcoming variants is still needed. In addition, an effective booster vaccination strategy created with consideration of the unique context of the country can help reduce the severity of the infection during breakthrough infection. Recently, Muik et al. reported that the Omicron breakthrough infection could have broader neutralization potential against emerging BA.2.12.1 and BA.4/BA.5 sublineages [4], while Zuo et al. (2023) also observed that the heterologous mRNA vaccination as a first or second booster dose following primary/booster vaccination with an inactivated vaccine regimen was significantly augmenting neutralization of emergent SARS-CoV-2 Omicron sub-variants, including BF.7, BQ.1.1, and XBB.1 [12].

In our current study, we found that the antibody level and the neutralizing ability against wild-type remained high in the population. Furthermore, the anti-spike antibody level displayed moderate correlation with the neutralizing ability of the antibody against B.1.1.529 variant and BA.2 Omicron subvariant. A high percentage of inhibition against the B.1.1.529 and BA.2 Omicron variants could be displayed in a wide range of anti-wildtype spike antibody levels. Normally, the detection of existing COVID-19 antibodies in a hospital determines mostly the antibody level to the spike of the wild-type strain, Wuhan-Hu-1, and that does not represent neutralizing antibody levels to other variants. Therefore, to evaluate the precise levels of binding antibodies to RBD of specific VoCs, a method such as an enzyme-linked immunosorbent assay using specific RBD of the variants is needed during the variant-dominant period. However, this method limits testing to special laboratories, and the available commercial anti-spike immunoassay displayed 65.2–69.6% detection rates for anti-Omicron antibodies, compared to live virus neutralization assays [20].

Unlike the antibody against wild-type, the neutralizing antibody to Omicron variants was correlated with the variant-dominant period. The percentage of inhibition against the Omicron variant from sera collected prior to the Omicron outbreak displayed lower than 20% inhibition against Omicron. One surprising finding to us was the great correlation between % inhibition against B.1.1.529 and BA.2 Omicron variants. The neutralization of Omicron variants was strong in sera of previously infected individuals receiving homologous and heterologous vaccines collected during the Omicron-dominant period. Corroborating this finding, Carazo et al. (2023) reported that, in previously infected individuals, the mRNA-based COVID-19 vaccines were found to provide 65%, 68%, and 83% protection against Omicron re-infection after the first, second, and third doses, respectively. In contrast, in individuals without prior infection, only 20%, 42%, and 73% protection was observed after the first, second, and third vaccinations. In addition, they suggested that no matter how many vaccinations were administered, previously infected individuals had 40–60% greater protection against reinfection [21]. Moreover, Omicron BA.2 is the parent lineage of the highly contagious Omicron subvariants, BA.4 and BA.5, and is more closely related to both subvariants. A recent study by Muik et al. reported that vaccinations with the SARS-CoV-2 wild-type S-based vaccines combined with breakthrough infections with the VOCs characterize the immunity patterns within the population. Therefore, an Omicron BA.2 breakthrough infection triggered neutralizing antibodies with broad neutralizing activity against BA.2 and all its three descendants—BA.2.12.1, BA.4, and BA.5 [4]. These data prompted our speculation that availability of heterologous vaccine regimens, especially prime/boost with different types of COVID-19 vaccines, along with breakthrough infections with the VOCs, is the reason for the rapid decline in overall global infections. Our study findings support the ongoing next-generation COVID-19 vaccine development against continuously emerging SARS-CoV-2 variants, so that the new vaccine will encourage those who have never been infected to become more resilient against ongoing variants.

We acknowledge that this study was conducted with a relatively small number of individuals in some groups, particularly recovery patients (Re, 25 subjects) and individuals who received a homologous vaccine combined with breakthrough infections (ReHo, 16 subjects). Due to the strict policy of COVID-19 vaccination for Thai people across the country, together with the severity of COVID-19, many recovered persons and most healthy people got vaccinated. As a result, the number of people infected with COVID-19 has been reported to decline rapidly. Consequently, it was difficult to find recovered but never-vaccinated subjects during the Omicron wave. Furthermore, the number of homologous vaccine recipients who have undergone breakthrough infections remains low due to the available vaccines and no strictly defined vaccine regimen in the country. During the Delta wave, the available vaccines were only inactivated and adenovirus-vector vaccines. Later, after the mRNA vaccines became available, people were interested in receiving the mRNA vaccine as a booster during the late Delta wave to the Omicron wave. The number of ReHo volunteers was limited. Unlike ReHo, however, the number of the ReHe individuals who recovered and received heterologous vaccines was larger. Due to the diverse vaccine regimens, a large number of ReHe volunteers would be needed to cover an equal number of individuals receiving different vaccine regimens. While comparison of a wide range of vaccine schemes would provide useful data for the selection of booster vaccination, the limited availability of vaccines and volunteers during the study period constrained our efforts. In addition, our own limited resources constrained the number of available samples in each scheme. Therefore, studies with larger sample sizes and group heterogeneity may be necessary in the future. Additionally, it is also critical to understand whether the durability of humoral responses against the Omicron XBB subvariants differs in groups receiving different priming and booster vaccination regimens. Certainly, supplementation with variant-characterizing methods such as Whole Genome Sequencing (WGS), or complete or partial spike-gene sequencing, would have made the results of recovery groups even more informative. Regrettably, the laboratories of the hospitals in our study did not conduct characterization of SARS-CoV-2 variants from infected persons, and all clinical specimens confirmed to contain SARS-CoV-2 have already been destroyed, following biosafety guidelines for handling of SARS specimens.

## 5. Conclusions

A breakthrough infection is the key for individuals who received COVID-19 vaccinations to develop high protective immunity against Omicron variants. Our observations robustly show that the breakthrough SARS-CoV-2 Omicron variant in individuals who received homologous or heterologous vaccines had a high level of neutralizing activity against B.1.1.529 and BA.2 parental lineages of recently circulating XBB Omicron subvariants. The protective level of immunity against closely related strains is highly correlated. Therefore, having immunity against strains related to the spreading variants will help prevent infection or reduce the severity of symptoms more than will relying on the immunity from the original SARS-CoV-2 vaccine alone. For persons who received only COVID-19 vaccinations without prior COVID-19 infection, it is absolutely necessary to get a booster from a next-generation COVID-19 vaccine developed against continuously emerging SARS-CoV-2 variants.

## Figures and Tables

**Table 1:** Sociodemographic characteristics of the participants.

Characteristics	Recovery Patients without Vaccination (*n* = 25)	Individuals with Full Doses Vaccination		Recovery Patients with Full Doses Vaccination		Total (*n* = 205)	*p*
		Homologous Vaccine (*n* = 46)	Heterologous Vaccine (*n* = 47)	Homologous Vaccine (*n* = 16)	Heterologous Vaccine (*n* = 71)		
Sex (*n* (%)) Male Female	10 (40.0) 15 (60.0)	18 (39.1) 28 (60.9)	9 (19.1) 38 (80.9)	6 (37.5) 10 (62.5)	20 (28.2) 51 (71.8)	63 (30.7) 142 (69.3)	0.603
Age [Median (interquartile range)]	44.5 (29.25–52.25)	40 (21–51)	34.5 (26.75–43.25)	43 (22–48)	26 (20–56)	28 (21–45)	0.001
Sample Collection windows	June–Nov 2021	June–Nov 2021	Oct 2021–Dec 2022	Nov 2021–Dec 2022	Nov 2021–Dec 2022	Jun 2021–Dec 2022	

**Table 2:** Antibody levels and neutralizing ability of five subject groups.

Terms [Median (Interquartile Range) *t* Value, (*p* Value) *]	Recovery Patients without Vaccination (*n* = 25)	Individuals with Full Doses Vaccination		Recovery Patients with Full Doses Vaccination		ANOVA *p* Value
		Homologous Vaccine (*n* = 46)	Heterologous Vaccine (*n* = 47)	Homologous Vaccine (*n* = 16)	Heterologous Vaccine (*n* = 71)	
Anti-spike IgG level (BAU/mL) *t* value (df) *p* value	257.342	905.192 2.382 (69) 0.02	1535.194 4.621 (70) <0.001	2342.190 5.218 (39) <0.001	2260.406 5.535 (94) <0.001	<0.001
% inhibition against wild-type *t* value (df) *p* value	73.581	80.984 1.299 (69) 0.198	93.520 4.342 (70) <0.001	93.246 2.958 (39) 0.005	95.600 7.219 (94) <0.001	<0.001
% inhibition against B.1.1.529 *t* value (df) *p* value	0.000	0.003 0.735 (69) 0.465	7.129 1.741 (70) 0.086	30.247 3.815 <0.001	59.587 8.599 (94) <0.001	<0.001
% inhibition against BA.2 *t* value (df) *p* value	0.000	1.599 1.048 (69) 0.298	21.387 2.925 (70) 0.005	39.565 4.256 <0.001	80.906 15.345 (94) <0.001	<0.001

* *p* value from Independent-samples *t*-test, recovery patients as reference group; df = degrees of freedom.

## Data Availability

Data supporting reported results may be provided on reasonable request to the corresponding author.

## Supplementary Materials

The following supporting information can be downloaded at: https://www.mdpi.com/article/10.3390/vaccines11071230/s1, Table S1: Percent inhibition against wild-type, Omicron B.1.1.529, and Omicron BA.2 in vaccine scheme of individuals with prior infection. Table S2: Percent inhibition against wild-type, Omicron B.1.1.529, and Omicron BA.2 in vaccine scheme of individuals with no history or evidence of infection.
