# Supplementary material for: Breakthrough SARS-CoV-2 Omicron Variant in Individuals Primed with Heterologous Vaccines Enhances Inhibition Performance of Neutralizing Antibody to BA.2 Parental Lineage
Source: Vaccines (Basel). 2023 Jul 11;11(7):1230. doi: 10.3390/vaccines11071230 (PMC10383937; doi:10.3390/vaccines11071230)
Supplement: Supplementary file 1 [file vaccines-11-01230-s001.zip › vaccines-2465663-supplementary.pdf]

Table S1. Percent inhibition against wild-type, Omicron B.1.1.529, and Omicron BA.2 in vaccine schemes of individuals with prior infection.

Vaccine Scheme * of individuals with prior infection	*n*	Serum collection period	% inhibition against Wild-type Median (interquartile range)	% inhibition against Omicron B.1.1.529 Median (interquartile range)	% inhibition against Omicron BA.2 Median (interquartile range)
2× Inactivated	6	Delta wave	94.73 (91.58–97.95)	0	0
	0	Omicron wave	ND	ND	ND
2–3× mRNA	1	Delta wave	98.14	0	0
	4	Omicron wave	95.8 (94.45–96.35)	88.98 (82.01–92.23)	96.12 (95.58–96.39)
2× Adeno-vector	5	Delta wave	97.9 (97.24–98.03)	0 (0–12.04)	0 (0–66.95)
	0	Omicron wave	ND	ND	ND
Inactivated +Adeno-vector	12	Delta wave	97.88 (96.95–97.99)	2.01 (0–44.81)	64.61 (0–86.21)
	6	Omicron wave	96.03 (94.92–96.80)	91.32 (72.24–92.80)	94.81 (91.73–96.55)
Inactivated +mRNA	4	Delta wave	98.14 (98.12–98.15)	41.62 (16.37–64.98)	86.45 (78.97–86.60)
	22	Omicron wave	96.59 (95.53–96.90)	74.91 (62.61–89.84)	94.43 (90.97–96.03)
Adeno-vector +mRNA	0	Delta wave	ND	ND	ND
	3	Omicron wave	93.96 (93.26–94.83)	89.45 (86.72–91.48)	95.56 (95.42–95.80)
Inactivated +Adeno-vector +mRNA	0	Delta wave	ND	ND	ND
	24	Omicron wave	96.285 (93.55–97.01)	78.07 (32.88–88.08)	94.82 (77.40–95.96)

* Note: Vaccine schemes named by order of prime and boost vaccine type; Inactivated vaccine, CoronaVac (Sinovac Biotech Ltd., Beijing, China), Covilo (Sinopharm, Beijing, China); Adeno-vector vaccine, Vaxzevria (AstraZeneca-University of Oxford, Oxford, UK) adenovirus-vector vaccine; mRNA vaccine, Comirnaty (Pfizer-BioNTech Inc., New York, USA), Spikevax (Moderna-NIAID, Cambridge, MA, USA); ND = not done.

Table S2. Percent inhibition against wild-type, Omicron B.1.1.529, and Omicron BA.2 in vaccine schemes of individuals with no history or evidence of infection.

Vaccine Scheme * of individuals without evidence of infection	*n*	Serum collection period	% inhibition against Wild-type Median (interquartile range)	% inhibition against Omicron B.1.1.529 Median (interquartile range)	% inhibition against Omicron BA.2 Median (interquartile range)
2× Inactivated	22	Delta wave	79.01 (48.20–86.43)	0	0
	0	Omicron wave	ND	ND	ND
2–3× mRNA	10	Delta wave	97.60 (97.24–97.96)	0	0
	2	Omicron wave	70.53 (66.99–74.08)	0.08 (0.04–0.11)	36.77 (35.33–38.19)
2× Adeno-vector	12	Delta wave	96.74 (89.07–98.01)	0	0
	0	Omicron wave	ND	ND	ND
Inactivated +Adeno-vector	13	Delta wave	97.73 (95.44–98.02)	0	0
	1	Omicron wave	39.02	0	20.56
Inactivated +mRNA	20	Delta wave	98.11 (97.94–98.24)	0 (0–9.53)	0 (0–82.79)
	3	Omicron wave	95.55 (80.15–96.08)	0 (0–46.37)	45.88 (33.51–71.09)
Adeno-vector +mRNA	9	Delta wave	97.98 (97.43–98.18)	0 (0–0.43)	0 (0–82.34)
	0	Omicron wave	ND	ND	ND
Inactivated +Adeno-vector +mRNA	0	Delta wave	ND	ND	ND
	1	Omicron wave	27.87	0	33.1

* Note: Vaccine schemes named by order of prime and boost vaccine type; Inactivated vaccine, CoronaVac (Sinovac Biotech Ltd., Beijing, China), Covilo (Sinopharm, Beijing, China); Adeno-vector vaccine, Vaxzevria (AstraZeneca-University of Oxford, Oxford, UK) adenovirus-vector vaccine; mRNA vaccine, Comirnaty (Pfizer-BioNTech Inc., New York, USA), Spikevax (Moderna-NIAID, Cambridge, MA, USA); ND = not done.
